# Regulation of Inflammatory Gene Expression in PBMCs by Immunostimulatory Botanicals

**DOI:** 10.1371/journal.pone.0012561

**Published:** 2010-09-03

**Authors:** Karen L. Denzler, Robert Waters, Bertram L. Jacobs, Yvan Rochon, Jeffrey O. Langland

**Affiliations:** 1 Biodesign Institute, Arizona State University, Tempe, Arizona, United States of America; 2 Department of Naturopathic Research, Southwest College of Naturopathic Medicine, Tempe, Arizona, United States of America; 3 Herbal Vitality, Inc., Sedona, Arizona, United States of America; Duke University Medical Center, United States of America

## Abstract

Many hundreds of botanicals are used in complementary and alternative medicine for therapeutic use as antimicrobials and immune stimulators. While there exists many centuries of anecdotal evidence and few clinical studies on the activity and efficacy of these botanicals, limited scientific evidence exists on the ability of these botanicals to modulate the immune and inflammatory responses. Using botanogenomics (or herbogenomics), this study provides novel insight into inflammatory genes which are induced in peripheral blood mononuclear cells following treatment with immunomodulatory botanical extracts. These results may suggest putative genes involved in the physiological responses thought to occur following administration of these botanical extracts. Using extracts from immunostimulatory herbs (*Astragalus membranaceus, Sambucus cerulea*, *Andrographis paniculata)* and an immunosuppressive herb (*Urtica dioica),* the data presented supports previous cytokine studies on these herbs as well as identifying additional genes which may be involved in immune cell activation and migration and various inflammatory responses, including wound healing, angiogenesis, and blood pressure modulation. Additionally, we report the presence of lipopolysaccharide in medicinally prepared extracts of these herbs which is theorized to be a natural and active component of the immunostimulatory herbal extracts. The data presented provides a more extensive picture on how these herbs may be mediating their biological effects on the immune and inflammatory responses.

## Introduction

Many complementary and alternative medicine (CAM) approaches and interventions are thought to exert activities by enhancing immune function. Botanical supplements are used worldwide with the expectation of boosting the immune response and reducing pathogen-associated symptoms [1]. For many individuals, especially those living in third world nations, herbal medicines are the only therapeutic resources available. In 1985, the World Health Organization estimated that perhaps 80% of the world population relied on herbs for primary health care needs [2], [3], [4], [5]. This widespread use of herbal medicines is not restricted to developing countries, as it has been estimated that 12-31% of patients in European countries utilize herbal medicines [2], [3], [4], [5], [6]. In the United States, nearly one out of every five Americans are currently using some form of CAM [3]. The *Journal of the American Medical Association* reported that between 1990 and 1997 the prevalence of herbal remedy use increased 380% in the United States [7]. Many animal studies and limited clinical studies have demonstrated the efficacy of botanical remedies, such as *Astragalus membranaceus*, related to immunopotentiating effects [8], [9], [10], [11], [12], [13], [14], [15], [16], [17], [18], [19], [20], [21]. Even with the widespread use and acceptance, a complete understanding of the biochemistry and mechanisms of action regarding herbal remedies has remained largely unknown [21], [22].

The actual medicinal relationship between plants and humans is historically far-reaching. Ancient civilizations, as early as 2,800 B.C., have utilized herbal methodologies that are only now being recognized by Western science [23]. Throughout the world, but especially in Europe and Asia, a tremendous change in the use and appreciation of herbal medicine has taken place. In Europe, estimates show that over $2 billion are spent on herbal products each year, and in Germany, at least 10% of the pharmaceutical market is herbal-based medicine [2], [4]. Herbal products represent a major economic force in the United States as well, with an estimated annual sales figure of $3.3 billion in 1998 [3]. The increased use of complementary and alternative medicine is slowly gaining support through increased studies by scientific researchers.

Traditional herbal medicine utilizes many botanicals thought to strengthen the body's resistance to illness through effects on the immune system. Animal and clinical studies suggest that many botanical extracts provide anticancer, pro-inflammatory, and anti-infective activities [8], [9], [10], [11], [12], [13], [14], [15], [16], [17], [18], [19], [20], [21]. Most herbs for the immune system are believed to act as general immune system stimulators and are thought to alter immune function through the dynamic regulation of cytokine expression [21]. Echinacea spp. is one of the top selling botanical medicines in North America. Though still mired in controversy, this herb apparently exerts its antiviral activity primarily through stimulation of the immune system [1], [24], [25]. Likewise, Astragalus spp. is one of the primary herbs in Chinese medicine used in Europe and Asia for antimicrobial activity [26], [27], [28]. Research suggests that Astragalus spp. extracts may have some direct antibacterial activity against both gram positive and negative bacteria while in vivo studies suggest positive effects on patients and animals infected with HSV, HIV, HBV or with viral myocarditis [8], [27], [29], [30]. The antiviral activity of Astragalus is thought to be mainly due to modulatory effects on the immune system, specifically through the induction of proinflammatory cytokines [21], [25], [31]. Though the effect on cytokine expression by many botanicals has been investigated, the number of cytokines measured has been limited. The most frequently studied cytokines are IL1, IL2, IL4, IL6, TNF and IFNgamma [21]. These cytokines are important pro-inflammatory mediators and regulators of the immune response, but provide a limited view of specific immunological activities.

Botanogenomics (also referred to as herbogenomics) is defined as the analysis of the biological effect of a particular botanical medicine through profiling of the affected genomic or proteomic alterations [20]. This methodology can provide a novel mechanistic understanding of the efficacy of a particular herbal preparation. The goal of this study was to identify and to provide additional insight into inflammatory genes induced in PBMCs following treatment with immunomodulatory herbal extracts. This data may provide a better understanding of how these herbs may be inducing the physiological responses observed in individuals following administration of various immunostimulatory herbal products. Though many studies focus on identifying the key active constituents present in a botanical extract, for this study the goal was to measure changes in genetic expression in cells treated with total herbal extracts which are commonly prescribed to individuals. Using microarray analysis, novel genes which may be involved in the immunostimulatory responses as well as other known biological effects of these herbs were identified. This provides a more complete understanding of the potential mechanism of action, effectiveness, and global genetic effect of these total botanical extracts on cells.

## Materials and Methods

### Botanical extract preparation

The following herbs were used in this study: Astragalus membranaceus, Sambucus cerulea, Andrographis paniculata, Echinacea angustifolia, Urtica dioica and Tylophora asmatica. Fresh wild-crafted 6–8 inch leafy tops of Urtica dioica were harvested from Western Washington, fresh Sambucus cerulea flowers were harvested from north central Washington State, and fresh Echinacea angustifolia roots were harvested from central Kansas. All fresh plants were shipped overnight express to the manufacturing facility for immediate validation and processing. Fresh plant material was validated using taxonomic keys. Dried Astragalus membranaceus root slices and dried Andrographis paniculata herb were purchased from Mayway Corporation (Oakland, CA), and dried Tylophora indica whole leaf was purchased from Hillgreen Company (Bangalore, India). Dried plant material was validated using herbal pharmacopoeia monographs. Fresh plants were mixed with distilled water/190 proof ethanol/glycerol at a ratio of 1∶2.5 (*Urtica* and *Sambucus*) or 1∶3 (*Echinacea*) [weight of botanical to volume of liquid], ground gently in a 1 gallon stainless steel Hamilton Beach blender, and the herb-liquid mixture was transferred to a clean amber colored gallon glass jar and sealed. Dried botanicals were ground in a 1 gallon stainless steel Hamilton Beach blender, transferred to a clean amber colored gallon glass jar, and a mixture of distilled water/190 proof ethanol/glycerol was added at a ratio of 1∶5 (weight of botanical to volume of liquid). Distilled water/190 proof ethanol/glycerol concentrations were as followed (*Astragalus* 74/26/0; *Andrographis* 69/26/5; *Sambucus* 58/37/5; *Urtica* 55/37/8; *Echinacea* 48/47/5; *Tylophora* 32/63/5). The mixtures were kept at room temperature for 2 to 6 weeks, followed by separation of the liquid portion from the solid herb portion using a mechanical press. The extracted liquid was filtered using unbleached paper filters, pooled, and dispensed in amber colored bottles. A sample of each extract was dried and all extracts were found to contain similar concentrations of non-volatile solutes (ranging between 62.4 – 136.8 mg/ml extract).

### Endotoxin quantitation

Endotoxin levels in the botanical extracts were determined using the Genscript ToxinSensor Chromogenic LAL Endotoxin Assay Kit. The manufacturer's protocol was followed and the endotoxin units/ml (EU/ml) determined by comparison to an *Escherichia coli* standard solution.

*PBMC isolation*: Human PBMCs were obtained from a commercial source (Lonza, Inc., Allendale, NJ) or from freshly drawn blood. Commercially obtained PBMCs were isolated from peripheral blood by apheresis and density gradient centrifugation. Cells were shipped on dry ice and stored in liquid nitrogen until use.

For fresh PBMCs, blood was collected by venipuncture into heparinized tubes. Whole blood was removed and added to an equal volume of balanced salt solution (0.01% D-glucose, 0.005 mM CaCl_2_, 0.098 mM MgCl_2_, 0.54 mM KCl, 14.5 mM Tris pH 7.6, 126 mM NaCl). Forty mls of blood/salt solution was layered on top of 10 mls Ficoll-Paque Plus (Amersham Biosciences) and centrifuged at 400×g for 40 minutes at 20°C. PBMCs were removed from the interface and washed in balanced salt solution. The collection of fresh PBMCs was approved by the Arizona State University and Southwest College of Naturopathic Medicine (SCNM) Institutional Review Boards. Participants were recruited from SCNM and blood draws performed at the SCNM Medical Center. All participants received and completed a written informed consent form prior to participation in the study.

### Cell treatment

PBMCs were resuspended in RPMI1640 with 10% fetal bovine serum (1×10^6^ cells/mL) in cell culture dishes. 1×10^7^ cells (in 10 mL media) were treated with 5 µL herbal extract or vehicle (ethanol) for the indicated times (3–18 hours). Treatment of cells with phorbol-12-myristate-13-acetate (PMA) (Calbiochem) was done at a final concentration of 50 nM.

*RNA isolation*: Following treatment (3–18 hours), total RNA was isolated and purified as per manufacturers protocol using an RNeasy kit (Qiagen). Briefly, the cell lysate was homogenized using a QIAshredder spin column. Any DNA contamination was eliminated by DNase digestion and the RNA was isolated using the RNeasy spin column.

### Microarray analysis

One µg of high quality total RNA was converted into double-stranded cDNA using the Affymetrix One-Cycle Target Labeling Kit using an oligo-dT primer containing a T7 RNA polymerase promoter. The double-stranded cDNA was cleaned using the Affymetrix GeneChip Sample Cleanup Module and biotin-labeled cRNA was made using the One-Cycle Kit. The biotin-labeled cRNA was purified using the GeneChip Sample Cleanup Module and quantitated on a spectrophotometer and analyzed on a 1% agarose TAE gel to check for a wide distribution of cRNA transcript sizes. Next, the cRNA was fragmented to approximately 35–200 bp by alkaline treatment (200 mM Tris-acetate, pH 8.2, 500 mM KOAc, 150 mM MgOAc) and fragmentation was verified on a 1% agarose TAE gel. Separate hybridization cocktails were made using 15 mg fragmented cRNA from each sample.

A total of 200 µL of each hybridization cocktail was hybridized to an Affymetrix Human Genome U133 Plus 2.0 array for 16 hours at 45°C in the Hybridization Oven 640. The Human Genome array measures the expression of over 47,000 transcripts and variants, including approximately 38,500 well-characterized human genes. Arrays were washed on the Affymetrix GeneChip Fluidics Station 450 according to manufacturer's instructions using a primary streptavidin phycoerythrin (SAPE) stain, subsequent biotinylated antibody stain, and secondary SAPE stain. After hybridization and washing, arrays were scanned using the Affymetrix GeneChip Scanner 3000 7G. Scanned images obtained by the Affymetrix GeneChip Operating Software (GCOS) v1.2 were used to extract raw signal intensity values per probe set on the array and calculate detection calls (absent, marginal, or present). Assignment of detection calls was based on probe-pair intensities for which one probe was a perfect match of the reference sequence and the other was a mismatch probe for which the thirteenth base (of the 25-oligonucleotide reference sequence) was changed. All raw chip data was scaled in GCOS to 150 to normalize signal intensities for inter-array comparisons. Reports generated in GCOS were evaluated for each array to check for eukaryotic controls, maximum scaling factors of approximately 3, at least 30% present calls (actual data ranged from 37–48%), and maximum GAPDH (3′/5′) ratios of 3.0. All microarray data is MIAME compliant and the full microarray expression data has been deposited and is available in the MIAME compliant database at: http://www.ncbi.nlm.nih.gov/geo/query/acc.cgi?acc=GSE19128.

### Quantitative real-time PCR

The microarray results were confirmed by quantitative real-time PCR (qPCR). Ten representative genes were selected from the microarray expression profiles and changes in the expression level confirmed using qPCR. qPCR was performed using iQSYBR Green Super mix (Bio-Rad). Briefly, cDNA template for each isolate was added to wells containing PCR reaction mix (iQSYBR Green Super mix and primers). Primers were obtained from SABiosciences. Reactions were done in a MiniOpticon real-time PCR detection system with CFX Manager software control (Bio-Rad).

### Flow cytometry analysis

THP-1 cells were grown in RPMI1640 with 10% FBS in nonadherent plastic dishes. Cells were treated with supernatant from either mock treated PBMCs or PBMCs treated with PMA or Astragalus extract for 18 hours. One million cells were washed and resuspended in PBS containing 1% BSA, 0.1% sodium azide, and 30% human AB serum (Innovative Research) followed by staining for 30 minutes with antibodies specific for CD11b (clone ICRF44), CD14 (clone MphiP9) or with isotype controls. Antibodies were purchased as PE-Cy7 or APC-Cy7 conjugates (BD, San Jose, CA). Propidium iodide was used to exclude dead cells from analysis. Cell fluorescence was analyzed from samples of 20,000 cells using a BD Bioscience FACSAria with FACSDiVa software.

## Results

Peripheral blood mononuclear cells (PBMCs) are often considered the industry standard for investigating many aspects of immunological response in a cell culture system. As a mixed population of T-lymphocytes, B-lymphocytes, natural killer cells, monocytes, macrophages and dendritic cells, PBMCs represent key cells involved in the genetic expression of cytokines leading to innate and adaptive immune and inflammatory responses. To investigate the cytokine response induced by immuno-stimulatory herbal extracts, PBMCs were treated with an herbal extract followed by microarray analysis on isolated cellular RNA. To date, the effect of immuno-stimulatory herbs has only been evaluated by observing the expression of a limited number of cytokines. Such studies can be misleading and do not provide a broad or complete understanding regarding the effect of the herbal extract on immune cells. In our studies, 1×10^7^ cells were treated with 5 microL herbal extract for 18 hours. This concentration was based on an average human prescribed dose in relation to the approximate total blood volume (2.5 mL extract per 5 L blood). Following incubation with the extract, total RNA was purified from the cells and mRNA expression levels were evaluated by microarray analysis using an Affymetrix platform.

**Figure 1:**
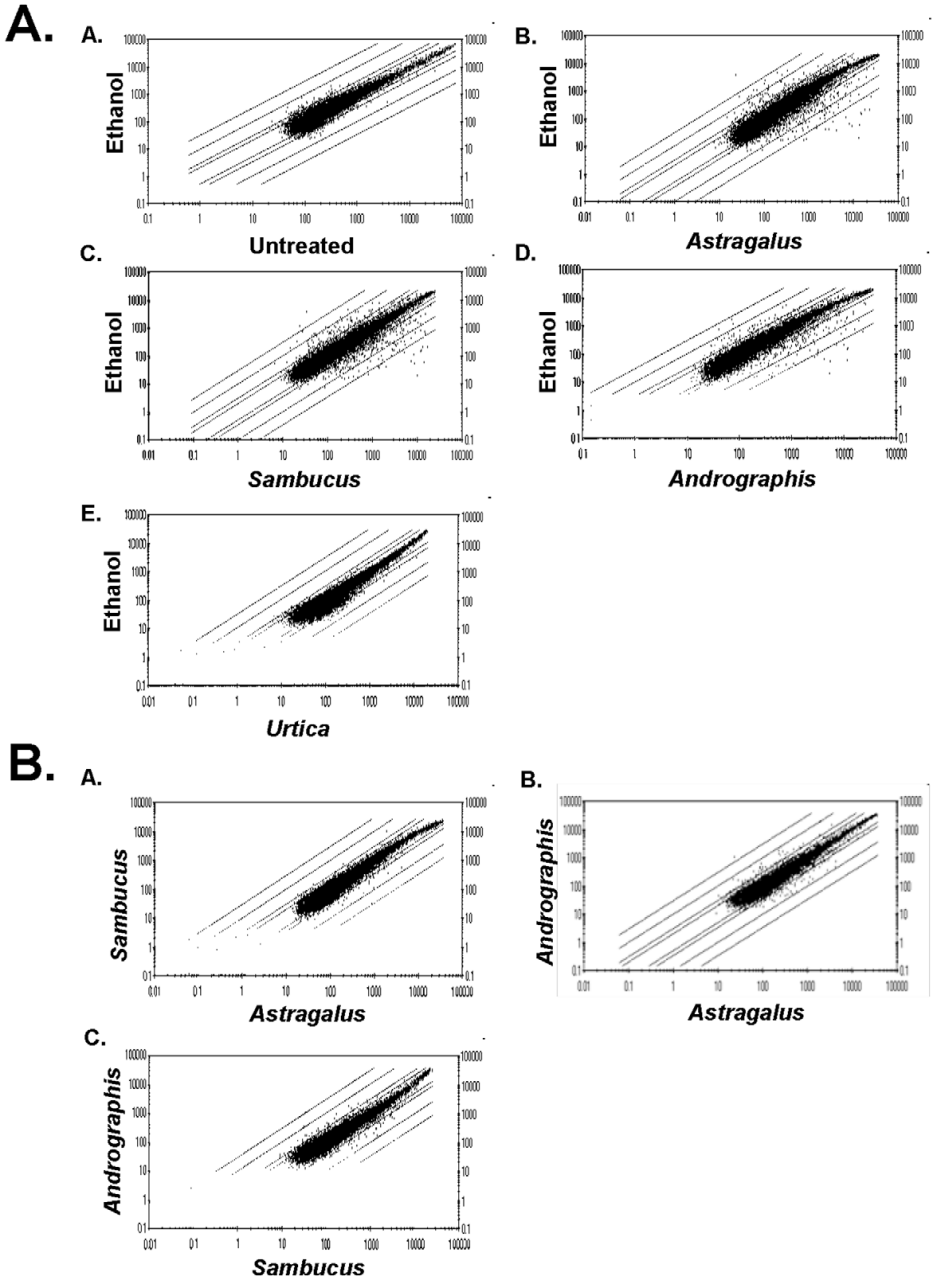
Scatter plot representation of botanical extract regulation of gene expression. Microarray analyzed gene data was plotted to compare gene expression differences between botanical and ethanol treatment of PBMCs (Part A). Each spot on the plots represents a specific gene. Only genes with present calls in both treatments are shown. The diagonal lines off the center represent 2-, 3-, 10-, and 30-fold levels of induction or repression of gene expression. Part B illustrates comparative analysis between different botanical treatments of PBMCs.

These studies were done using herbal extracts historically used to boost immune activity in patients, including *Astragalus membranaceus* (Milk-Vetch Root, Huang qi), *Sambucus cerulean* (Blue Elderberry), and *Andrographis paniculata* (India Echinacea, King of Bitters). Since these extracts were prepared in an aqueous vehicle containing ethanol and glycerol, PBMCs were treated with identical concentrations of ethanol and glycerol in distilled water (ethanol) as a control. Our initial method to measure modulation in gene expression was done using scatter plot analysis. Treatment of PBMCs with the vehicle solution had almost no effect on gene expression when compared to untreated PBMCs (Figure 1A, Plot A). However, as shown in Figure 1A, treatment of PBMCs with *Astragalus* extract led to the alteration of expression of many cellular genes (compare diffuse scattering of Figure 1A, Plot A to Plot B). In this figure, the diagonal lines off the center represent 2-, 3-, 10-, and 30-fold levels of induction or repression of gene expression. With *Astragalus* treatment, the expression of several hundred cellular genes was altered, many to levels greater than 30-fold relative to ethanol treatment alone (Figure 1A, Plot B). The *induction* of gene expression was greater than the level of gene *repression* in regard to the relative fold-change and number of genes altered (146 genes induced with 58% induced greater than 10-fold vs 118 genes repressed with 21% repressed greater than 10-fold). A similar scatter plot appearance was observed after treatment with *Sambucus* (Figure 1A, Plot C). After treatment of PBMCs with *Andrographis*, similar fold changes of induction/repression were observed, but fewer genes appeared to be affected (Figure 1A, Plot D). Since all three of these immuno-stimulatory herbal extracts produced relatively similar scatter plot profiles, an extract prepared from the immuno-suppressive herb, *Urtica dioica,* was studied. Treatment of cells with *Urtica* led to very limited or low level changes in cellular gene expression (Figure 1A, Plot E). The lack of change in gene expression after treatment with *Urtica* argues that the alteration in gene expression by the immuno-stimulating extracts (e.g., *Astragalus)* was not due to a non-specific effect of *any* botanical extract, but instead was a legitimate effect due to specific components present in the immuno-stimulating herbal extracts. It was not surprising that the *Urtica* extract did not alter gene expression since the PBMCs were from a reportedly healthy individual and immune-suppressive effects would likely not be observed. This effect has been observed previously where oral administration of an *Urtica* extract had no effect on cytokine expression in a healthy individual and only repressed cytokine expression following lipopolysaccharide induction [32].

Although PBMCs represent the primary immune cells involved in cytokine production, other cell types may also be involved in the immune and inflammatory responses associated with these botanical extracts. Therefore the intestinal cell line, Caco2 cells, were used and changes in gene expression measured following treatment with the botanical extracts. Results demonstrated no dramatic changes in gene expression following treatment with any of the botanical extracts (data not shown). Since PBMCs are key cells involved in cytokine production, these results suggest that the active constituents in these botanicals may interact with cells in the PBMC population leading to changes in cytokine expression.

The microarray results from treated PBMCs were confirmed by quantitative real-time PCR (qPCR). Ten representative genes were selected from the microarray expression profiles and changes in the expression level confirmed using qPCR. All ten genes tested using qPCR showed comparable gene expression levels as those obtained in the microarray analysis (Table 1). From our previous experience with microarray studies, significant variations between expression levels determined by microarray analysis or qPCR have not been observed [33].

**Table 1:** Quantitative real-time PCR (qPCR) verification of microarray data.

Gene	Microarray	qPCR
IL8	+89.9	+111.4 (+6.8)
IL1beta	+107.66	+137.2 (+7.1)
Oncostatin M	+38.97	+18.4 (+4.2)
IL10	+8.02	+4.9 (+2.3)
Vanin 1	+117.33	+147.0 (+7.2)
CCL4	+8.39	+16.0 (+4.0)
Thrombomodulin	+13.23	+14.9 (+3.9)
TLR5	−4.54	−4.0 (−2.0)
CXCL9	−25.0	−13.0 (−3.7)
IFNgamma	0	+1.4 (+0.5)

Select genes which were induced or repressed based on microarray data were verified using qPCR. Microarray values are listed as the fold-change following *Astragalus membranaceus* treatment relative to ethanol treatment. qPCR values are listed as the fold change (outside parentheses) and the change in the cycle threshold (C_t_) (inside parentheses) following *Astragalus* treatment relative to ethanol treatment (+  =  induction, −  =  repression).

**Figure 2:**
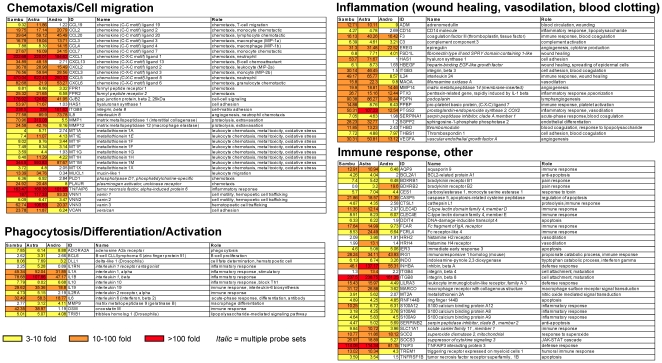
Host gene expression regulated by *Astragalus membranaceus* treatment of PBMCs. Genes were sorted based on a threefold (*P*<0.01) or greater level of induction for *Astragalus* treated PBMCs for 18 hours (Astra column). Only genes involved in the immune/inflammatory response are shown. Changes (n-fold) in expression level relative to those of ethanol-treated cells are shown within each box. Red boxes represent genes induced 100-fold or higher, orange boxes represent genes induced 10 to 100-fold, and yellow boxes represent genes induced 3 to 10-fold. Additional botanical treatments include *Sambucus cerulea* (Sambu column) and *Andrographis paniculata* (Andro column).

In order to compare the gene expression profiles altered by the herbs, comparative scatter plots were done between the immunostimulatory herbs. As shown in Figure 1B (Plot A), the genes altered by *Sambucus* and *Astragalus* were very similar as indicated by a lack of diffuse scattering of the genes. Treatment with *Astragalus* did lead to additional changes in gene expression as compared to *Sambucus*, indicated by a few genes induced 3–10 fold specifically by *Astragalus.* These *Astragalus* specific alterations occurred toward unique genes as well as to genes regulated by both *Astragalus* and *Sambucus* but were induced to a higher expression level following *Astragalus* treatment (Figure 2). More differences in gene expression were observed when comparing *Astragalus* to *Andrographis* or *Sambucus* to *Andrographis* (Figure 1B, Plot B and Plot C, respectively). Again, the genes altered by these comparative herbs were similar, but each herb also induced a unique subset of genes (data not shown and Figure 2). This data suggests that immuno-stimulating herbs may consistently affect a common set of genes while also having additional unique effects on gene expression.

The cellular genes induced and repressed after treatment with *Astragalus* were identified as (a) those genes which lie outside the scatter plot profile of untreated vs. ethanol treatment (Figure 1A, Plot A) and (b) those genes which were altered in gene expression at least 3-fold compared to ethanol treatment alone. From our experience and others on microarray studies, a fold-change of 2.5 or higher is typically considered significant; so limiting our fold-change to 3-fold or higher provides strong confidence in the validity that the expression of these genes was truly being altered [33]. Following treatment of PBMCs with *Astragalus* extract, approximately 150 genes were induced (data not shown). At least 65% of the genes that were induced by *Astragalus* are known to have functions involved in the immune/inflammatory response. Figure 2 (Astra column) shows the immune/inflammatory genes induced upon treatment with *Astragalus* extract.

Previous studies on *Astragalus* have suggested that treatment leads to (a) increased phagocytic activity of macrophages, (b) an increase in proinflammatory cytokines IL1, IL6 and TNF, and (c) an increase in levels of lymphocyte stimulatory IL2 and IL2 receptor expression [14], [21], [34], [35]. Immune and inflammatory uses of *Astragalus* include antimicrobial therapy [26], [27], [28], the treatment of leukemia and lung cancer [35], [36], [37], and wound healing [35], [38]. Based on literature and anecdotal evidence, *Astragalus* treatment may be associated with increased risk of bleeding, and blood pressure lowering effects [39], [40]. The genes induced in our study by *Astragalus* treatment agree well with these previous studies and the physiological effects of the herb on the body. In PBMCs, IL1alpha, IL1beta, IL6 and TNFalpha induced/interacting proteins (e.g., TNFAIP6) were highly induced, as well as genes involved in immune cell stimulation, chemotaxis, extravasation, and maturation/differentiation (Figure 2). In addition, genes involved in increased risk of bleeding (e.g., thrombomodulin, monoamine oxidase), regulation of blood pressure (e.g., adrenomedullin, prostaglandin endoperoxide synthase 2 [Cyclooxygenase-2], monoamine oxidase), and wound healing (epiregulin, fibronectin type III, heparin-binding EGF-like growth factor, interleukin 24) were identified (Figure 2). The effect of *Astragalus* on the expression of most of these genes has not been previously shown, and may provide new insight into the physiological effects of this herb.

In support of the comparative scatter plots (Figure 1B), a very similar set of genes regulated by the *Astragalus* extract was also regulated by the *Sambucus* extract (Figure 2, compare Astra column to Sambu column). This suggests that similar physiological activities are present in both botanical extracts, however, the active components may still be unique (i.e., conservation of function with diverse chemical constituents). Treatment of cells with the *Andrographis* extract also altered many genes in common with *Astragalus*, but overall, the level of induction of genes regulated by Andrographis was lower (Figure 2, Andro column).

**Figure 3:**
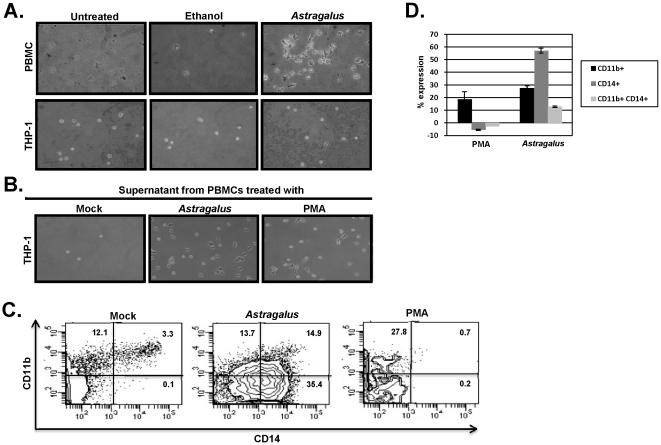
*Astragalus membranaceus* treatment of PBMCs led to monocyte maturation. Part A) PBMCs (upper panels) or the monocyte cell line THP-1 (lower panel) were untreated, treated with ethanol (25%), or treated with *Astragalus* extract for 18 hours. Following treatment, unattached cells were removed and the remaining attached cells photographed. Part B) PBMCs were untreated or treated with *Astragalus* extract or PMA for 18 hours. After 18 hours, the cell culture media was removed and cells pelleted by centrifugation. The cell-free culture media was added to THP-1 cells for an additional 24 hours. Following treatment, unattached cells were removed and the remaining attached cells photographed. Part C) Cell-free media from mock-, Astragalus, or PMA-treated PBMCs was added to THP-1 cells and incubated in uncoated plastic dishes. After 48 hours, cells were washed and stained with fluorochrome-conjugated antibodies specific for CD14 and CD11b followed by flow cytometry analysis. Part D) Cell-free media obtained from PBMCs isolated from two patients were used to treat THP-1 cells and analyzed for CD14 and CD11b expression as in Part C. Values indicated represent total CD14 cells, total CD11b cells and CD14/CD11b double-positive cells. Data was normalized to mock-treated samples.

Monocytes represent a significant percentage of cells present in the PBMC population. They can mature into macrophages or differentiate into dendritic cells (DCs) upon migration into tissues. Monocytes that have matured into macrophages become more adherent to tissue culture surfaces and increase expression of CD11/CD18 integrins [41], [42]. To test for monocyte maturation, PBMCs were treated with Astragalus extract and assayed for the effect on cell attachment. As shown in Figure 3A (upper panels), treatment of PBMCs with Astragalus extract led to an increase in adherent cells. Quantitatively, the number of adherent cells was increased approximately 15-fold. These results suggest that treatment of PBMCs with an immuno-stimulating herb, such as Astragalus, not only increases the transcription of immune related genes, but that these gene products are inducing immune cell maturation and/or differentiation. It is known that IL4 and GM-CSF mature monocytes towards DCs and that IL6 directs monocyte maturation primarily towards macrophages [43]. Upon treatment of PBMCs with Astragalus extract, IL6 expression was highly up-regulated (Figure 2), whereas IL4 expression and GM-CSF was unchanged. These results suggest that the monocyte maturation observed upon treatment with Astragalus extract may be directed toward a macrophage lineage. This is in agreement with previous data in which the isolated APS polysaccharide from Astragalus was shown to induce macrophage activation [44]. To investigate if this monocyte maturation was due to a direct effect of the extract on monocytes, monocyte to macrophage maturation following treatment with Astragalus extract was tested using the THP-1 monocyte cell line. Treatment of these cells with the Astragalus extract led to no significant increase in adherent cells as compared to untreated or ethanol treated cells suggesting the lack of a direct effect of the extract on monocyte to macrophage maturation (Figure 3A, lower panels). The maturation of monocytes observed in the PBMC population may be linked to cytokines released from other responder cells present in the PBMC population (e.g., T helper cells). To test for this, the cell-free culture media from untreated or Astragalus treated PBMCs (24 hours post treatment) was added to THP-1 cells. As shown in Figure 3B, an increase in adherent cells was observed in the THP-1 cells treated with the cell-free media from Astragalus treated PBMCs but not from untreated PBMCs. As a control, the cell-free media from PMA-treated PBMCs was added to THP-1 cells. Similar increases in adherent cells were observed using either cell-free media from PMA or Astragalus treated cells (Figure 3B). These results suggest that the Astragalus induced monocyte maturation in the PBMC population was due to a secondary effect following the release of cytokines from responder cells.

Maturation of THP-1 cells by cell-free media was further monitored by the expression of CD14 antigen and by expression of the β2 integrin component, CD11b, both of which are upregulated when this promonocytic cell line differentiates into mature monocyte/macrophage-like cells [45]. Incubation of THP-1 cells with cell-free media from mock-treated PBMCs resulted in 15.4% of cells expressing CD11b while incubation with cell-free media from either Astragalus- or PMA-treated PBMCs resulted in 28.5% or 28.6%, respectively, of cells expressing CD11b. It has been previously shown that the presence of adhesion-associated molecules on monocytes does not necessarily correlate with adherence to a substrate, but that adherence requires intracellular signaling which conformationally activates the adhesion molecule [46]. Mock-treated THP-1 cells may therefore express an inactive form of CD11b that does not mediate adherence (Figure 3C). Incubation of THP-1 cells with cell-free media from mock- or PMA-treated PBMCs resulted in 3.4% and <1% of cells expressing CD14, respectively. Incubation with cell-free media from Astragalus-treated PBMCs resulted in expression of CD14 in 50.3% of the cells. In addition, extracts from *Astragalus*-treated PBMCs caused a 4.5-fold increase in the percentage of double-positive cells expressing both CD14 and CD11b as compared to mock-treated PBMCs. Figure 3D shows averaged levels of expression of total CD14 or CD11b positive cells and CD14+CD11b+ double positive cells, normalized to mock levels, from PBMCs isolated from two different patients. Although cell-free media from both *Astragalus*- and PMA-treated PBMCs induced maturation as measured by cell adhesion and an increase in percentages of total CD11b+ cells, only media from *Astragalus*-treated PBMCs induced expression of CD14.

**Figure 4:**
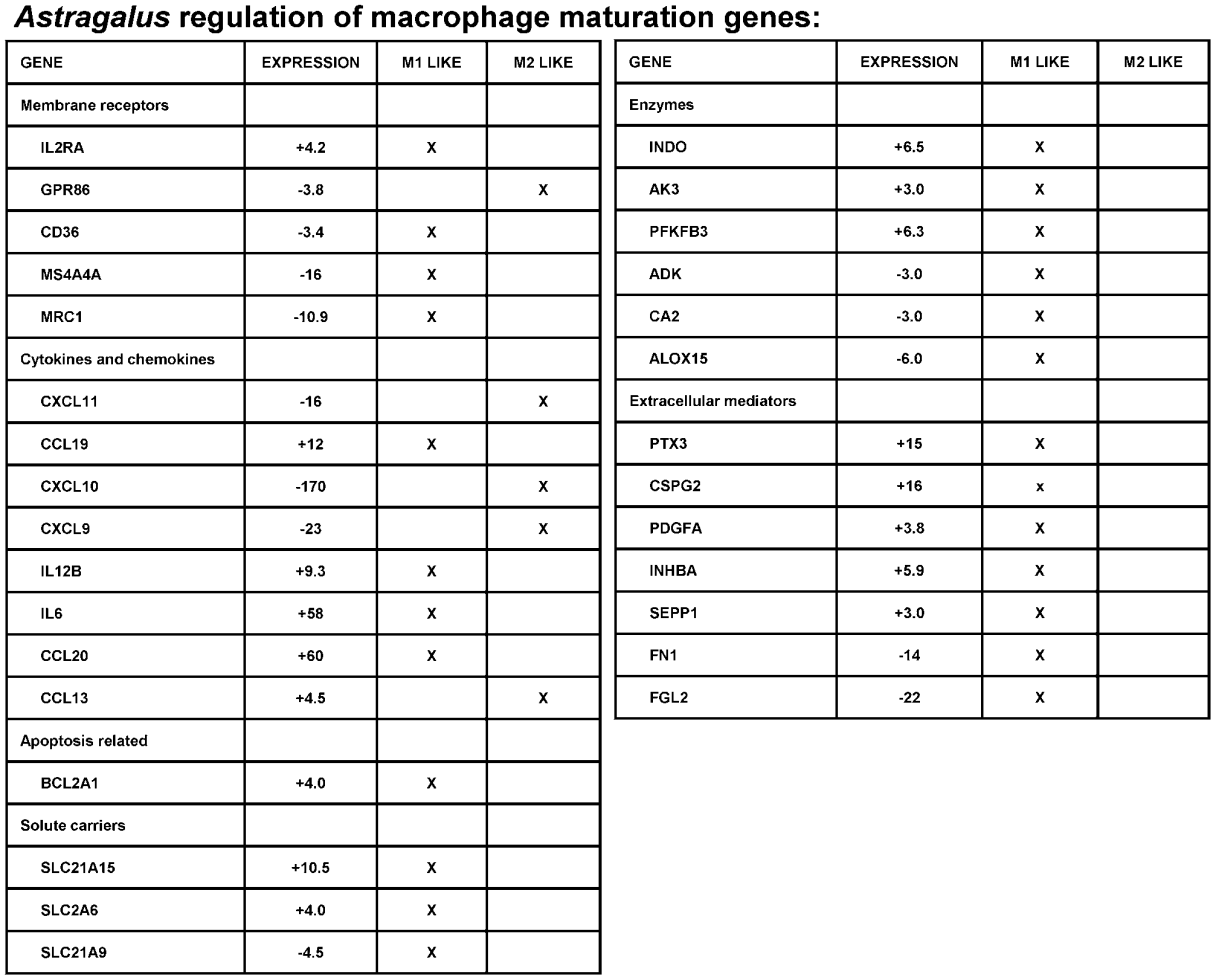
Transcriptional profiling of M1/M2 macrophage polarization induced by *Astragalus membranaceus.* Human genes involved in defining M1/M2 polarization are shown [47]. The transcriptional regulatory effect of each gene following treatment of PBMCs with *Astragalus* extract is indicated (‘Expression’ column), with positive values representing increased expression, and negative values representing repressed expression. The ‘X’ (in the ‘M1 like’ or M2 like' column) indicates that the change in gene expression (induced or repressed) following *Astragalus* treatment was similar to that previously observed following monocyte maturation to either an M1-like or M2-like macrophage polarization.

In general, the cytokine gene profile induced following treatment with *Astragalus* was not strongly directed toward either a Th1 or Th2 response, but rather a more generalized or preparative immune/inflammatory response (see Figure 2). Most genes involved in defining a Th1 or Th2 response remain unchanged (eg. Th1: IFNgamma, IL18, SOCS1, STAT1, CSF2; Th2: IL13, IL4, IL5, ICOS). However, gene expression profiles have been defined regarding human monocyte to macrophage maturation and polarization toward M1 or M2 phenotypes [47]. As shown in Figure 4, the gene expression profile of *Astragalus* treated PBMCs follows an M1 polarization (with both induced and repressed genes) with 83% of the genes defining the polarization of M1/M2 macrophage matching that of an M1 lineage. In agreement with this, COX-2 which was highly induced following treatment with *Astragalus* (Figure 2), has long been associated and induced in M1 macrophages [48]. Likewise, COX-1, which is up-regulated in M2 macrophage [47], was slightly repressed following *Astragalus* treatment (data not shown). These results suggest that *Astragalus* treatment matured monocytes toward an M1 polarization.

**Figure 5:**
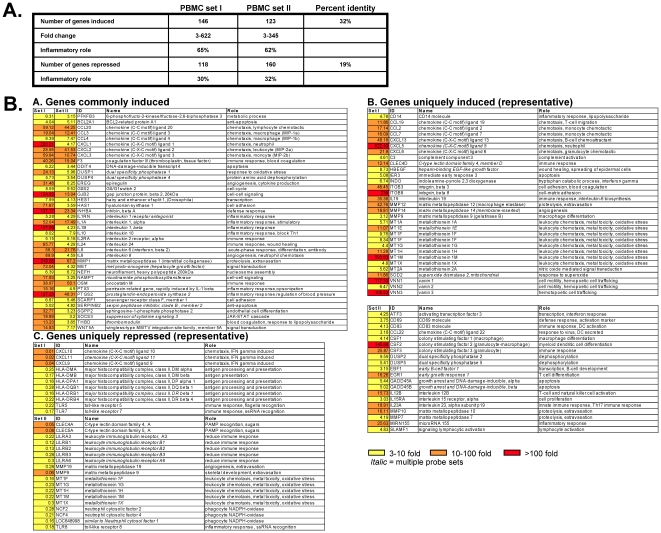
Different PBMC isolates led to similar changes in gene expression following treatment with *Astragalus membranaceus.* Commercially available (PBMC set I) or fresh PBMCs (PBMC set II) were treated with *Astragalus* extract for 18 hours. Genes were sorted based on a threefold (*P*<0.01) or greater level of induction or repression relative to ethanol-treatment. Part A represents the overall comparison of genes regulated between PBMC set I and PBMC set II. For Part B, specific immune/inflammatory genes commonly or uniquely altered in expression following *Astragalus* treatment of PBMC set I and PBMC set II are shown. Changes (n-fold) in expression level relative to those of ethanol-treated cells are shown within each box. Red boxes represent genes induced 100-fold or higher, orange boxes represent genes induced 10-100-fold, and yellow boxes represent genes induced 3-10-fold.

Since the microarray data presented thus far represents treatment of a single population of PBMCs from a commercial source (Lonza, Inc. [identified as PBMC set I]), we wanted to confirm that similar gene modulation would occur in fresh human PBMCs. Fresh PBMCs were obtained and purified as described in the Materials and Methods (identified as PBMC set II). Following treatment of these cells with *Astragalus* extract, 123 genes were found to be induced (Figure 5A, PBMC set II), giving a similar response as that observed with the commercially obtained PBMCs, PBMC set I (146 genes induced). Like PBMC set I, the genes induced in PBMC set II primarily had an immune/inflammatory role (65% vs. 62%, respectively). Although many of the inflammatory genes induced were different between the two PBMC populations, over 30% of the induced genes were identical. This level of identity is high given that the PBMCs were from different individuals and obtained from fresh vs. frozen. The genes commonly induced are likely many of the key players involved in the known physiological responses to *Astragalus* including those involved in immune cell activation, differentiation and chemotaxis (eg. CCL3, CCL4, CXCL1, CXCL2, CXCL3, IL1alpha, IL1beta, IL6 and IL8), as well as wound healing and blood coagulation (eg. coagulation factor III, prostaglandin endoperoxide synthase 2, thrombomodulin, and IL24) (see Figure 5B, panel A).

Genes that were induced in only one of the PBMC sets are likely related to human to human genetic variations and/or physiological state of the individual at the time of the blood draw. Many of the uniquely induced genes represented functional families of genes. For example, in PBMC set I following treatment with *Astragalus*, several metallothioneins, vanins, and integrins were induced (Figure 5B, panel B). In PBMC set II, these gene families remained primarily unchanged, however gene families including colony stimulating factors, dual specific phosphatases, and growth arrest and DNA-damage inducible were induced. Notably, PBMC set II also led to the unique induction of CD69, early growth response 1 and microRNA 155 which are all key proteins involved in the inflammatory response (Figure 5B, Panel B). Previous studies demonstrate that considerable natural variation in gene expression levels exists within and among human populations [49]. When they compared individuals, gene expression variations occurred in an estimated 83% of genes. Genes which are differentially expressed among populations may be particularly relevant as candidates for disease, or in our studies, for differences in the immune response.

We also identified over 100 cellular genes which were repressed in expression levels after treatment with *Astragalus* (Figure 5A). In this data set, the majority of the genes (approximately 70%) were *not* directly involved in the inflammatory/immune response, but were genes involved in metabolic pathways such as lipid and carbohydrate metabolism (e.g. fructose-1,6-biphosphatase 1, fucosidase, lipase A, and alpha galactosyltransferase 1). This data supports the concept that these herbs are involved in the induction of a systemic inflammatory and immune response. Again, when PBMC set I and PBMC set II were compared, similar numbers and percentages of genes were repressed (Figure 5A), but for those genes involved in the inflammatory response, specific gene families to each PBMC set were repressed. For PBMC set I, several major histocompatability complex class II genes were repressed (Figure 5B, panel C). For PBMC set II, repressed gene families included leukocyte immunoglobulin receptors, C-lectin domain family members, neutrophil cytosolic factors and metallothioneins (Figure 5B, panel C). At this time, it is difficult to speculate on what role the suppression of these inflammatory gene families may play in the physiological effects of *Astragalus.*

**Figure 6:**
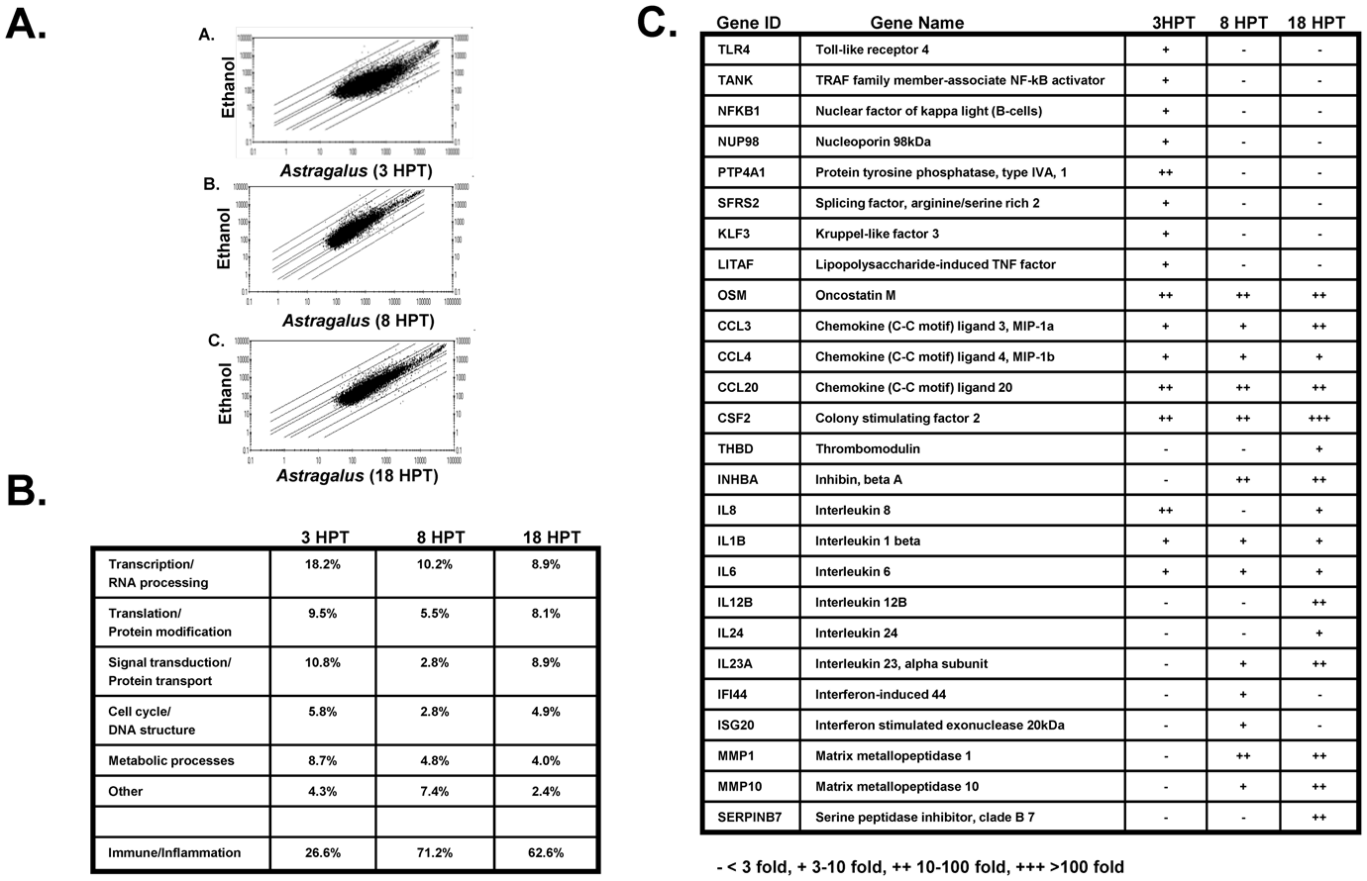
Temporal regulation of gene expression in PBMCs following *Astragalus membranaceus* treatment. PBMCs were treated with *Astragalus* extract for 3, 8 or 18 hours. Microarray analyzed gene data was plotted to compare gene expression differences between botanical and ethanol treatment of PBMCs (Part A). Each spot on the plots represents a specific gene. Only genes with present calls in both treatments are shown. The diagonal lines off the center represent 2-, 3-, 10-, and 30-fold levels of induction or repression of gene expression. Part B represents the roles and overall comparison of genes induced at 3, 8 and 18 hours post *Astragalus* treatment. Part C lists representative genes induced at 3, 8 and/or 18 hours post *Astragalus* treatment.

In order to further understand the temporal effect of *Astragalus* treatment on immune cells, PBMCs were treated with the botanical extract and microarray analysis performed on RNA samples prepared at 3, 8 and 18 hours post treatment. Figure 6A shows scatter plot representation of gene expression levels of ethanol treated PBMCs in comparison to cells treated with the *Astragalus* extract for the indicated times. As shown, the cellular transcriptional response to the herbal extract was rapid with a dramatic induction/repression of several hundred genes by 3 hours post treatment (Figure 6A, panel A). By 8 and 18 hours post treatment, fewer genes were induced/repressed, but still represented 100–200 genes (Figure 6A, panels B and C). The induction of genes by *Astragalus* was biphasic with different families of genes being induced at different times post treatment. As shown in Figures 6B and 6C, at 3 hours post treatment, there was a predominance of genes (approximately 40%) involved in transcriptional, translational and signal transduction processes with only 26.6% of genes having an inflammatory role. However, by 8 and 18 hours post treatment, approximately 70% of the genes induced were involved in the immune/inflammatory response (Figure 6B and 6C). The immune genes which were induced at 3 hours post treatment typically continued to be induced through 18 hours, including various chemokines (CCL3, CCL4, CCL20) and interleukins (IL1beta and IL6) (Figure 6C). Interestingly, there are differences in the immune/inflammatory genes induced between 8 and 18 hours post treatment. Genes involved in the type I interferon response, such as interferon-induced 44 and interferon stimulated exonuclease 20 kDa, were induced only at the 8 hour time point (Figure 6C). Likewise, several interleukins were only induced at the 18 hour time point, including IL12B and IL24 (Figure 6C). Since PBMCs contain a variety of immune cells, these temporal effects may be due to constituents present in the herbal extract interacting with (a) responder cells which subsequently induce/release cytokines and lead to transcriptional responses in other effector cells, or (b) cells which induce cellular receptors/signal transduction processes which, *after* expression, can respond to additional herbal constituents leading to inflammatory gene induction.

**Figure 7:**
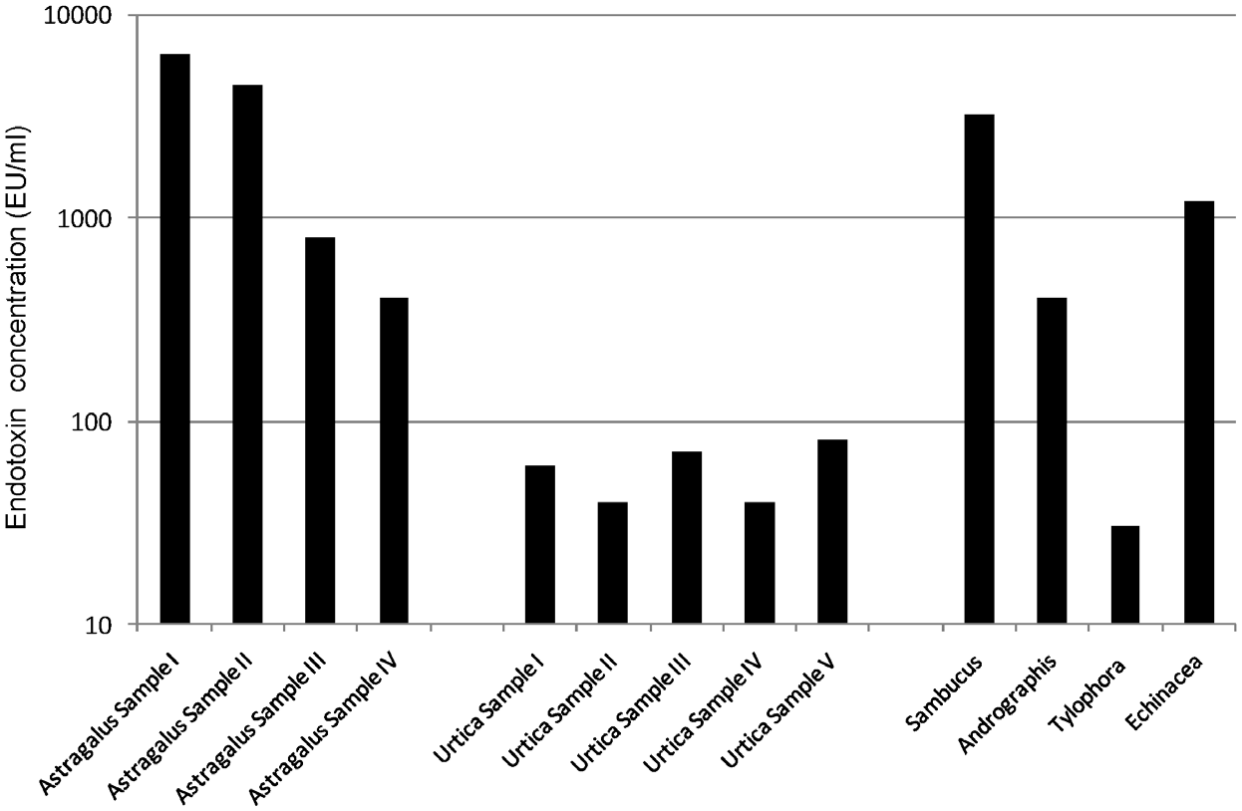
Endotoxin concentration present in botanical extracts. Endotoxin concentrations in the botanical extracts listed were measured using a modified Limulus Amebocyte Lysate assay. Concentrations (endotoxin units/ml; EU/ml) were determined by comparison to an *Escherichia coli* standard solution. For *Astragalus* and *Urtica*, multiple extract preparations were prepared at different times using multiple lots of plant material obtained from the supplier (Samples I–IV and Samples I–V, respectively).

Lipopolysaccharides (LPS) are major components of the outer membrane of Gram-negative bacteria and highly potent activators of the innate immune response. The interaction of LPS via CD14 with cells, such as macrophages, granulocytes, and dendritic cells, leads to the synthesis of a multitude of inflammatory mediators [50], [51]. However, strong inflammatory responses to LPS can be hazardous and may lead to endotoxin shock and death [50], [51]. Therefore the presence of LPS or endotoxin contamination in pharmaceutical drugs and medicines is a common concern. The herbal extracts used in these studies were assayed for the presence of endotoxin using a modified Limulus Amebocyte Lysate kit. As shown in Figure 7, herbal extracts historically reported and used to stimulate the immune response (*Astragalus membranaceus, Sambucus cerulea, Andrographis paniculata, Echinacea angustifolia* and *Glycyrrhiza glabra*) [10], [52], [53], [54], [55]) had highly elevated levels of endotoxin (>100 EU/ml). Those herbal extracts historically used to repress immune activity (*Urtica dioica* and *Tylophora asmatica*) [56], [57]) had much lower endotoxin levels (<100 EU/ml). This correlation between activity and endotoxin concentration may suggest that the presence of LPS was not due to contamination of the extract, but instead was a common constituent of the immune-stimulatory extracts and as such, may play a role in immune regulatory activity. In order to confirm that the LPS present in the extracts was not due to contamination during harvesting or extract preparation, multiple extracts of *Astragalus* and *Urtica* were prepared using different lots of plant material from the suppliers. As shown in Figure 7, all the extracts of *Astragalus* had high endotoxin levels (>100 EU/ml) and all the extracts of *Urtica* had low endotoxin levels (<100 EU/ml). This further supports that the presence of LPS in the botanical extracts was not due to exogenous bacterial contamination during harvesting, handling, or extract preparation, and instead was likely due to the lysis of endophytic bacteria which colonize the internal tissue of the plants in a mutualistic and asymptomatic fashion. Of the nearly 300,000 plant species that exist on earth, it is believed that each individual plant is host to one or more bacterial endophytes [58], [59], [60]. Endophytes are defined as endosymbionts that live within a plant without causing any apparent disease. Endophytes are not only found in the roots and in the rhizosphere, but are often found to colonize the intercellular spaces and have been isolated from all plant compartments, including seeds [60]. A vast number of these endophytes, including *Rhizobium sp.* which has been isolated from *Astragalus*, are Gram-negative bacteria [59]. Based on this, it is expected that many botanical extracts would contain LPS due to lysis of these bacterial endophytes.

## Discussion

The goal of this study was to obtain a more complete understanding of the regulation of cellular gene expression by various immuno-stimulatory herbs. Since the personal use and prescribing of total herbal extracts has increased dramatically in recent years, this study sought to measure changes in cytokine gene expression in cells treated with a total botanical extract. Even though these assays were done in a cell culture system, the results may aide in understanding the physiological effects thought to be associated with administration of these extracts to individuals. Administration of these herbal extracts to individuals is typically done orally, leading to sublingual and intestinal uptake of the active constituents into the bloodstream. It is unknown if these constituents are altered in the enteric environment, however sublingual administration allows direct diffusion into the bloodstream. Once in the bloodstream, these constituents likely interact with PBMCs leading to cytokine production. Although many studies focus on identifying the key active constituents present in an herbal extract, this was beyond the scope and focus of this study. Validation and confidence in the data obtained from the microarray expression studies was supported in our results where (1) many of the genes altered in expression were detected by multiple probe sets present on the microarray chips and demonstrated similar levels of expression between these probes (Figure 3), (2) many genes altered in expression were similarly altered by different herbs used in this study (Figure 3), (3) different cellular samples had a significantly similar set of genes altered in expression (Figure 5), (4) quantitative real-time PCR of select genes demonstrated similar changes in gene expression as obtained in the microarray study (Table 1), and (5) only those genes with a 3-fold or greater (*P*<0.01) change in expression were included in our analysis.

*Astragalus* is a staple of Traditional Chinese Medicine being one of the oldest medicinal herbs listed in the material medica of Chinese herbal medicine [61]. Chinese herbalists have used *Astragalus* to supposedly help the human body resist disease [62]. It is the most commonly used herb in Chinese medicine. Modern herbalists utilize *Astragalus* primarily as an immunostimulant to prevent common infection and aid in the recovery following infection [62]. Several animal studies and double-blinded, randomized human pilot studies have demonstrated an effect on cytokine production and immune cell activation following ingestion of *Astragalus* extracts [8], [9], [10], [11], [12], [13], [14], [15], [16], [17], [18], [19], [20], [21]. Historically, the biological activities associated with *Astragalus* have been accounted for, at least in part, to several constituents present in the herb including flavonoids, saponins, and polysaccharides [61], [63]. Our data suggests that LPS present in the *Astragalus* extract may be a key component as well. *Astragalus* led to the induction of IL1alpha, IL1beta, IL6, and IL8 which likely leads to an early proinflammatory response. These proinflammatory cytokines are known to be induced by LPS and were upregulated by all three immune stimulatory herbal extracts tested [64]. The Th1-specific cytokines, IL2, TNFalpha, and IFNgamma, were not upregulated in either of the samples and may suggest that an infectious agent may need to be present to further drive the immune response. Surprisingly, TNFalpha, which is known to be induced by LPS, was not detected after *Astragalus* treatment [64]. This may suggest that the type(s) of LPS present in the extract does not induce TNFalpha or that other components of the extract act to inhibit the induction of TNFalpha. An alternate explanation may be that the LPS-inducible regulatory cytokine IL10 which was detected in both PBMC samples acted to down regulate or prevent induction of IL2, TNFalpha and IFNgamma which may be important in protection from necrotic injury. However induction of the alpha chain of the IL-2 receptor was observed in both samples suggesting a readiness to induce a Th1 response in the advent of infection. Additionally, *Astragalus* has been described as being able to redirect an underlying Th2 immune response toward a Th1 response as seen in patients with chronic viral infection or cancer [8], [37]. Our microarray data supports this idea since we observed the modulation of multiple genes fitting an M1-type macrophage profile to be up-regulated in our PBMCs [47]. The ability of *Astragalus* to shift the immune response toward a Th1 phenotype is supported by previous *in vivo* studies of viral infection which require a Th1 response for resolution [8], [27], [29], [30].

Based on the characterized functions and activities with *Astragalus*, our microarray data is in agreement with the historical uses of *Astragalus* and may provide additional insights into how this herb may be inducing putative physiological effects. As well as those cytokines previously described as being up-regulated by *Astragalus,* we found additional cytokines induced by this herb, many of which play a role in chemotaxis and extravasation (e.g. CCL2, CCL7, CCL19, CCL20, CXCL1, CXCL5, CXCL6, CXCL13, formyl peptide receptor 1 and 2, MMP1, MMP12 and mucin-like 1). A number of these cytokines can be induced by LPS in macrophages from healthy patients (CCL19, CCL20, CXCL1, CXCL13, MMP1) and is associated with genes responsive to NF-κB [65]. However, many LPS-responsive genes with or without NF-κB binding sites were not induced by *Astragalus* and suggests alternate modes of gene regulation by the extract. Based on our data, *Astragalus* appears to induce a generalized and/or preparative state regarding stimulation of the immune response, which agrees with the suggested physiological effects of this herb. Of note, multiple MIPs (macrophage inflammatory proteins) were induced, including MIP-1α, MIP-1β, MIP-2α, and MIP-2β (also termed CCL3, CCL4, CXCL2, CXCL3, respectively). CCL3, CCL4, and CXCL2 are known to be induced by LPS and may suggest a role for this *Astragalus* component in the mobilization of polymorphonuclear leukocytes for innate defense [65].

In addition to these effects on the immune response, *Astragalus* has been described as having an effect on wound healing and blood pressure modulation. In regards to the role of *Astragalus* with wound healing, no cellular proteins had previously been described as being involved in this physiological response. It has recently been shown that *Astragalus* extracts significantly accelerated cutaneous wound healing by stimulating basal cell growth in the wound area and promoting angiogenesis [38]. From our microarray studies, several putative proteins were identified which could affect wound healing. These include adrenomedullin, fibronectin type III and SPRY domain containing 1-like, heparin binding EGF-like growth factor, vascular endothelial growth factor (VEGF), hyaluronan synthase 1, sphingosine-1-phosphate phosphatase 2, and IL24. In addition, several genes which were induced (epiregulin, matrix metallopeptidase 14, podoplanin, VEGF, thrombospondin-1) are key players in angiogenesis. This agrees with recent reports which suggest that *Astragalus* promotes angiogenesis and induces VEGF expression [38], [66]. Most of the above genes are not known to be responsive to LPS with the exception of VEGF and possibly sphingosine 1-phosphate phosphatase 2 which both contain NF-κB binding sites in their promoters [67], [68]. Additionally, LPS is known to adversely impair wound healing [69]. This suggests that alternate components of *Astragalus* are likely mediating the wound healing and angiogenic effects and may be modulating the activities of LPS. Regarding blood pressure modulation, prostaglandin-endoperoxide synthase 2 (COX-2), adrenomedulin (ADM) and monoamine oxidase were all induced and are known to be involved in vasodilation. LPS induces both COX-2 and ADM and may be an active component associated with blood pressure modulation [65], [70]. The vasodepressor role of the COX-2 gene product has been shown to be regulated and dependent on the activity and ratio of both COX-1 and COX-2 [71]. Notably, treatment of PBMCs with the *Astragalus* extract led to a high induction of COX-2 transcription while slightly inhibiting COX-1 gene expression possibly supporting a vasodepressor effect. Similarly, several proteins were induced which can affect blood coagulation, including coagulation factor III, integrin β3, and thrombomodulin, and these genes have not been shown to be LPS-induced. Interestingly, thrombomodulin can prevent LPS induction of inflammatory molecules by the inhibition of NF-kB signaling [72]. Botanical components, as well as the natural presence of LPS in the *Astragalus* extracts may together be responsible for the induction of genes involved in angiogenesis, modulation of blood pressure, and blood clotting [73], [74], [75]. Further studies are required to determine the effects of other components present in the *Astragalus* extracts that may modulate the activities of LPS in putative physiological roles.

*Sambucus* extract, like *Astragalus*, is primarily prescribed for its immune-modulating, antioxidant and antiviral activities. Limited studies with *Sambucus* have demonstrated its ability to significantly increase the production of IL1beta, IL6, IL8, and IL10 [24], [53]. These cytokines were similarly upregulated in our microarray studies, as well as many other cytokines. For the antioxidant activity associated with *Sambucus*, we found that superoxide dismutase 2 was highly induced and could be associated with this activity. Both *Sambucus* and *Astragalus* induced a very similar set of cytokines, and typically, with a very similar level of induction. Though not well characterized, the immunologically active constituents of *Sambucus* likely include flavonoids and anthocyanins [76], as well as LPS as determined in this study. This data suggests that these divergent herbs have similar effects on immune cells, though possibly through distinctly different effectors.

*Andrographis* extract has traditionally been used for its anticancer and immunomodulatory activity [55], [77]. In our microarray results, *Andrographis* induced many immunostimulatory genes similar to *Astragalus* and *Sambucus*, but was substantially different, typically with the expression of many fewer genes being altered. This may suggest that *Andrographis* does have immunostimulatory activity, but the effect on the immune cells may be greatly reduced or directed toward a more specific response. Unfortunately, a direct comparative study between these herbs has never been done. Interestingly, *Andrographis* has been suggested to decrease blood pressure [78] as does bacterial LPS [79], and like *Astragalus*, it led to the induction of vasodilators, including adrenomedullin and prostaglandin-endoperoxide synthase 2 (COX-2).

*Urtica* is typically used for rheumatologic conditions due to its anti-inflammatory and immunosuppressive activities [57]. In our study, this herb was effective in demonstrating that the genes induced by the immunostimulatory herbs were specific and not due to the exposure of any botanical extract to the immune cells. Treatment of cells with the *Urtica* extract led to almost no changes in cellular gene expression. This supports a previous clinical study where administration of *Urtica* to a healthy individual had no effect on cytokine gene expression, including TNF, IL1, IL4, IL6 and IL10 [32]. However, following immunostimulation in these subjects, *Urtica* was able to reduce the expression of these cytokines, and this activity is likely due to the ability of *Urtica* to inhibit activation of the transcription factor, NF-κB [80].

The level of expression is an important aspect of gene action. It is clear that reduced or increased gene expression can influence many quantitative traits., Immunostimulatory herbs may alter the expression of similar genes, but may also have dramatically different effects on the physiological response through significant differences in gene expression levels or specific differences in genes regulated. Many botanical extracts, including those in this study, have been used for their antimicrobial activity [31]. Although some of this activity may be due to a direct antimicrobial effect from these herbs, much of this activity may be due to the immunostimulatory activity which we have described.

*Astragalus* treatment led to the induction of specific proinflammatory cytokines over the entire course of treatment (from 3 to 18 hours). However, there was also a distinct temporal response in gene expression. Early after treatment, many of the genes induced were involved in signal transduction processes and RNA/protein modification. Following this initial response, the subsequently induced genes were primarily involved in amplifying the inflammatory response. This type of temporal inflammatory response has previously been observed in mice treated with immunostimulants. Han, *et al*. found that immuno-stimuli led to the early induction of several genes, including cytokines, which subsequently led to the secondary induction of gene expression. Though not completely understood, this type of temporal activation is thought to be associated with amplified, perpetuated and sustained inflammatory processes [81]. This type of response would fit with the physiological activity for which *Astragalus* is utilized.

It has been previously described that considerable natural variation in gene expression levels exists within and among human populations [49]. In our study, using PBMCs from two distinctly different individuals, over 30% of the genes induced by *Astragalus* were commonly altered in expression. Based on genetic diversity, this level of similarity was notable and likely represents those genes characteristically induced by *Astragalus* in immune cells. As expected, many of these conserved genes are involved in the proinflammatory response as well as wound healing, decreasing blood pressure and increasing the risk of bleeding, (including various cytokines and interleukins, hyaluronan synthase 1, sphingosine 1 phosphatase 2, thrombomodulin, epiregulin, monoamine oxidase, prostaglandin-endoperoxide synthase 2 [COX-2]). As expected with human diversity, each individual sample also induced a specific subset of genes. The vast majority of these genes were involved in the immune/inflammatory response and likely represent individual responsiveness to the botanical immune stimuli. For example, PBMCs from one individual induced high levels of the gene family of colony stimulating factors (CSF1, CSF2 and CSF3) following treatment with *Astragalus.* These genes were unaltered in expression in the other PBMC set. Given that these CSFs aid in stimulating the production of various white blood cells, they are key factors in the proinflammatory response. Since these genes were expressed with individuality, they likely affect the individual responsiveness and effectiveness to *Astragalus* in regards to the immune response.

As mentioned, all the extracts used in this study are representatively the same as those regularly prescribed to patients by licensed physicians in the complementary and alternative medicine field. With four different immuno-stimulatory herbs tested (*Astragalus, Sambucus, Andrographis,* and *Echinacea*), all were found to have relatively high levels of LPS present in the extract. Importantly, much lower levels of LPS were detected in extracts from herbs historically used for immuno-suppression (*Urtica*, and *Tylophora*). Our data supports that this LPS was not due to contamination during harvesting, handling or extract preparation, but instead was due to symbiotic endophytic bacteria present in the plants. Since a vast number of botanical endophytic bacteria are Gram-negative [59], the LPS present in the extracts was likely obtained by lysis of these bacterial cells. Although identifying the active constituents present in these botanical extracts was not the focus of this study, our data supports a role of LPS in regulating the immunological response. Future studies will focus on deciphering the specific types and role of LPS in these cellular responses.

All extracts utilized in this study were prepared by Herbal Vitality, Inc. (Sedona, Arizona, USA). This company was chosen since they are a source for herbal products used by licensed practitioners in the field. This company ensures identification of all plant species and prepares highly active and reproducible herbal extracts according to traditional and accepted extraction methods used in the complementary and alternative medicine field. This is an important aspect of this study since all the extracts used in this study are representatively the same as those regularly prescribed to patients by licensed physicians. Previous studies on immune-stimulating herbs have commonly focused on purifying active components which are free of endotoxins. For this study we wanted to evaluate the cellular response to immune-stimulatory herbal extracts in the context of extracts representative of those which are prescribed to patients. Since many of these prescribed immunostimulatory extracts appear to contain LPS, likely from the lysis of endophytic bacteria, this research represents a more physiologically relevant study to investigate the potential cellular response following exposure to the whole herbal extract.

As mentioned above, various components from these immuno-stimulatory herbs have been shown to have immuno-stimulatory roles and act to induce cytokine expression. Based on the known roles of LPS, it is expected that the LPS present in these extracts is influencing immune gene expression as well. The cytokines, IL1alpha, IL1beta, IL6, and IL10 were expected to be induced by LPS, however we did not observe induction of other typically-induced cytokines like TNFalpha or MCP-2. However, LPS structure and activity varies greatly between different bacteria and LPS structures on endophytic bacteria have not been well characterized [50]. Indeed, *Rhizobium sp.* has been shown to establish a symbiotic relationship with the roots of *Astragalus* [82], [83]. Unlike LPS derived from enterobacteria, which activates immune cells via the transmembrane Toll-like receptor 4 (TLR4), the structurally different LPS from *Rhizobium* acts through TLR2 and TLR6 [50], [51], [82]. Although TLR2 and TLR6 are thought to be similarly involved in immune cell stimulation, the gene expression profiles and cellular response may be different than activation through TLR4. Endophytic bacteria have been identified in the tissues of every living plant known [58], [59], [60]. Since botanical extracts have been used by humans for centuries for the treatment of various medical conditions, components from these bacteria may be involved in the historical activities associated with these herbs. Recent studies support that botanical endophytic microbes synthesize active antiviral, anticancer, antioxidant and immunosuppressive compounds [58]. LPS from these bacteria may represent one more active group of compounds involved in the activity of these herbs. Future studies will focus on the isolation and characterization of the LPS from the endophytic bacteria and on determining the specific role in regulating gene expression and the inflammatory response.

In conclusion, though microarray analysis does not provide conclusive proof that the genes induced by the immunostimulatory herbs are responsible for the physiological effects observed in humans following administration of the various herbs, it does provide insight into putative genes and directions for future research. It should be noted that many herbs, including *Astragalus*, are typically considered safe, even at high doses and prolonged use [84]. Our data supports that *Astragalus* treatment induced genes involved in a strong, generalized proinflammatory response. This immune response may be responsible for much of the antimicrobial activity associated with *Astragalus.* However, care and further investigation should be considered regarding prolonged, uninterrupted use of some herbs, such as *Astragalus*, which may lead to physiological effects associated with sustained inflammatory activity.
